# FgMob1 is involved in mitotic exit and regulates the development, abiotic stress response, and pathogenicity of *Fusarium graminearum*

**DOI:** 10.3389/fpls.2026.1871251

**Published:** 2026-06-11

**Authors:** Zenghong Luo, Qihao Xu, Xintong Wu, Xuzhao Mao, Usama Amin, Yakubu Saddeeq Abubakar, Wenhui Zheng, Zonghua Wang, Huawei Zheng

**Affiliations:** 1 Fujian Key Laboratory of Conservation and Sustainable Utilization of Marine Biodiversity, College of Geography and Oceanography, Minjiang University, Fuzhou, China; 2 Institute of Plant Protection, Fujian Academy of Agricultural Sciences, Fuzhou, China; 3 State Key Laboratory of Agricultural and Forestry Biosecurity, Fujian Agriculture and Forestry University, Fuzhou, China

**Keywords:** abiotic stress, FgMob1, *Fusarium graminearum*, mitotic exit, pathogenicity

## Abstract

*Fusarium graminearum* is a plant pathogenic fungus that causes wheat scab. This pathogen is distributed worldwide and produces deoxynivalenol, which significantly affects humans and animals. Mob1 belongs to the MOB (Mps One Binder) family, whose members are present in a wide variety of eukaryotes, and is a core component of the MEN (mitotic exit network) pathway, regulating the mitotic exit process in yeast. However, the roles of Mob1 in pathogenic fungi remain poorly understood. In this study, we investigated the roles of FgMob1 in the development and pathogenicity of *F. graminearum*. Functional analyses showed that FgMob1 is important for vegetative growth, conidiation, ascospore formation, DON production, and pathogenicity. Subcellular localization results revealed that FgMob1 localizes to the spindle pole bodies. Furthermore, the average number of nuclei was significantly increased in the hyphae and conidia of the *FgMOB1* deletion mutant, suggesting that FgMob1 is involved in MEN. Additionally, FgMob1 plays a significant role in the response to abiotic stress, deletion of the *FgMOB1* gene affected sensitivity to cell wall, plasma membrane and oxidative stresses. In summary, this study demonstrates that FgMob1 is involved in MEN and is required for vegetative growth, asexual and sexual development, abiotic stress response, and pathogenicity in *F. graminearum*.

## 1 Introduction

*Fusarium graminearum* is a plant pathogenic fungus that causes Fusarium head blight (FHB), one of the most significant and destructive diseases affecting wheat crops worldwide (Buttar et al., 2024; Wu et al., 2025). *F. graminearum* has been listed among the top 10 plant pathogenic fungi globally (Dean et al., 2012). This fungus enters host cells through natural openings, forms lobate appressoria and infection cushions (Bushnell et al., 2003; Jiang et al., 2019), and then produces exocellular enzymes and effectors to inhibit host immunity (Chen et al., 2019; Li et al., 2025; Shang et al., 2025). After infection, the fungus spreads rapidly in wheat and produces the trichothecene mycotoxin deoxynivalenol (DON), which threatens food and feed safety globally, causing serious harm to the health of humans and livestock (Buttar et al., 2024; He et al., 2025; Kang et al., 2026). Numerous mechanisms control the cell cycle to ensure correct cell division, understanding the precise regulatory mechanisms of the cell cycle in pathogenic fungi is essential for the prevention and control of these pathogens. MEN is required for the release and activation of the Cdc14 phosphatase, which promotes cyclin-CDK inactivation (Visintin et al., 2003). In budding yeast, the Mob1-Dbf2 complex directly phosphorylates Cdc14 on serine/threonine residues adjacent to its nuclear localization signal, driving Cdc14 translocation from the nucleolus to the cytoplasm during exit from mitosis (Mohl et al., 2009). Recent studies have shown that septins, FgCdc14, and Cdc2A are involved in cell cycle regulation, morphogenesis, and pathogenicity in *F. graminearum* (Li et al., 2015; Chen et al., 2016; Jiang et al., 2016). Additionally, mitotic exit mediated by the small GTPase FgTem1 and its GAP FgBub2-FgBfa1 is crucial for the pathogenicity of *F. graminearum* (Miao et al., 2023).

The mitotic exit network (MEN) is required for exit from mitosis during the budding process. Mutants arrest in late anaphase with elongated spindles, segregated chromosomes, high CDK activity, and fail to initiate cytokinesis (Mccollum and Gould, 2001). This network includes Tem1, Cdc15, Mob1, Dbf2, and Cdc5 (Bardin and Amon, 2001; Lee et al., 2001). In budding yeast, the MEN is key to normal mitosis during the budding process (Mccollum and Gould, 2001). In the fission yeast *Schizosaccharomyces pombe*, a similar pathway, termed the septation initiation network (SIN), regulates the initiation of cytokinesis at the end of anaphase (Krapp and Simanis, 2008).

The Mob (Mps one binder) proteins are a small family of highly conserved, non-catalytic proteins found in a wide variety of eukaryotes (Luca and Winey, 1998), first identified in yeast (Luca and Winey, 1998). In unicellular organisms such as yeast, the MOB family includes Mob1 and Mob2 (Luca and Winey, 1998), while in multicellular organisms such as flies, there are at least four different MOBs (He et al., 2005). In humans, there are as many as seven different MOB proteins (Gundogdu and Hergovich, 2019). In animals, Mobs act as adaptors in Hippo signaling, an intracellular signal-transduction pathway that restricts growth, impacting the development and homeostasis of animal organs, and are possibly linked to cancer and other diseases (Gundogdu and Hergovich, 2019; Duhart and Raftery, 2020). Mob1 encodes a functionally important 314-amino acid protein that contains no known structural motifs and is necessary for completion of mitosis and maintenance of ploidy in yeast. It was identified from a two-hybrid screen as a protein that binds Mps1p, a protein kinase essential for spindle pole body duplication and mitotic checkpoint regulation (Luca and Winey, 1998). The crystal structure of yeast ScMob1 has been reported, comprising both the conserved C-terminal core and the variable N-terminal region (Mrkobrada et al., 2006). Within the N-terminal region, three novel structural elements are observed (an α-helix denoted H0, a strand-like element denoted S0, and a short β strand denoted S-1) which are functionally relevant. In humans, hMOB1A and hMOB1B are two LATS-binding proteins that may function as tumor suppressors in human cancer cells (Chow et al., 2010). In plants, MsMob1 plays a key role during the reproductive pathway in *Medicago sativa* (Citterio et al., 2005). In *Aspergillus fumigatus*, MOB-mediated regulation of SIN signaling is a key factor in echinocandin-induced hyperseptation (Thorn et al., 2024). In the rice blast fungus *Magnaporthe oryzae*, MoMob1 is required for vegetative growth, conidiation, and virulence (Feng et al., 2021), and is also involved in MEN. However, the roles of Mob1 in other pathogenic fungi are largely unknown.

There are three Mob proteins in the genome of *F. graminearum* (Liu et al., 2021). FgMob2 interacts with the NDR kinase FgCot1 and is critical for polarity, fungal development, cell wall organization, lipid metabolism and virulence in *F. graminearum* (Liu et al., 2021). The STRIPAK complex component FgMob3 orchestrates cell wall integrity signaling to govern fungal development and virulence in *F. graminearum* (Chen et al., 2023). However, the functional role of FgMob1 in *F. graminearum* remains unclear. Our previous study showed that mitotic exit mediated by the small GTPase Tem1 is essential for the pathogenicity of *F. graminearum* (Miao et al., 2023). However, the functional role of other components of the MEN in *F. graminearum* has not been elucidated. To further identify the role of MEN in *F. graminearum*, we analyzed the functions of FgMob1 in this study. Phenotypic analysis showed that FgMob1 localizes to the spindle pole bodies and is a component of MEN. Further analyses revealed that it regulates the vegetative growth, sexual and asexual reproduction, DON production, and virulence in *F. graminearum*. Moreover, disruption of MEN in *F. graminearum* affects the fungal response to abiotic stress.

## 2 Materials and methods

### 2.1 Fungal strains, and culture media

The wild type (WT) PH-1 and all the mutant strains used in this study are listed in **Supplementary Table 1**. PH-1 and all mutants were cultured on three different media (minimal medium (MM), complete medium (CM) or starch yeast medium (SYM) at 28 °C for 3 days (Luo et al., 2025). Sexual reproduction and conidiation were assayed according to previous report (Zheng et al., 2015; Zhang et al., 2023). 0.02% SDS, 200 μg/mL calcofluor white (CFW), and 0.03% H_2_O_2_ were added to CM for stress sensitivity test (Guo et al., 2026).

### 2.2 Fungal transformation and generation of gene deletion mutants

Protoplast preparation and fungal transformations were performed according to standard protocols (Hou et al., 2002). A split-marker approach (Catlett et al., 2003) was used to generate *FgMOB1* gene deletion mutants using the primers listed in **Supplementary Table 2**. Candidate transformants were screened by PCR and further confirmed by Southern blot.

### 2.3 Construction of pFgMob1-GFP vector and complementation

For the pFgMob1-GFP fusion vector, the primers *FgMOB1*-CF and *FgMOB1*-CR listed in **Supplementary Table 2** were used to amplify the full-length *FgMOB1* gene, including its native promoter from wild type PH-1 genomic DNA. The construct was cloned into the pKNTG2 vector using a One Step Cloning Kit (C113-02-AB, Vazyme Biotech Co. Ltd, Nanjing, China), and the chimeric DNA was verified by sequencing. The pFgMob1-GFP plasmid was transformed into the protoplasts of the ∆*Fgmob1* mutant for complementation.

### 2.4 Plant infection and DON production assays

Infection assays of PH-1 and mutants on flowering wheat heads, wheat coleoptiles and corn silks were conducted (Zheng et al., 2021; Luo et al., 2025), mycelial plugs of the PH-1, mutants and complemented strains were inoculated on the flowering wheat heads and corn silks, and observed symptoms 14 days or 7 days after inoculation, respectively; conidia of the PH-1, mutants and complemented strains were inoculated on the wheat coleoptiles and observed symptoms 7 days after inoculation. For DON production assays, all strains were grown in liquid trichothecene biosynthesis induction (TBI) medium at 28 °C for 7 days in the dark; the liquid was used for the DON production assay, while the mycelia were quantified after drying (Zheng et al., 2021). All experiments were repeated three times.

### 2.5 Live cell imaging

A Nikon A1R laser scanning confocal microscope system (Nikon, Tokyo, Japan) was used for live cell fluorescence imaging of *F. graminearum*. For hyphal localization assays, a mycelial block from SYM agar containing the leading hyphae was excised, placed upside down on a coverslip, and observed directly. GFP and mCherry excitations were set at 488 nm and 561 nm, respectively. To examine the number and distribution of nuclei, the FgHistone1-mCherry fusion protein was constructed as described previously (Miao et al., 2023) and then transformed into the protoplasts of PH-1 and ∆*Fgmob1* strains, respectively. The transformants were observed under a confocal microscope as described above.

### 2.6 Bioinformatics and statistical analyses

The amino acid sequence of FgMob1 in *F. graminearum* was identified by BLAST search using the amino acid sequence of MoMob1 protein from *Magnaporthe oryzae* in the fungal database (https://fungidb.org/fungidb/app). All experiments were carried out in three independent replicates, and the results were statistically evaluated using one-way ANOVA for multiple comparisons, followed by Tukey’s test. Differences were considered statistically significant at *P* < 0.05. The statistical program GraphPad Prism 5.0 (GraphPad Software, Inc., USA) was used to draw graphs.

### 2.7 Accession numbers

*F. graminearum* (FgMob1-XP_011315949.1).

*Fusarium oxysporum* (FoMob1-XP_018233055.1).

*Fusarium verticillioides* (FvMob1-XP_018743091.1).

*Neurospora crassa* (NcMob1-XP_956516.3).

*Magnaporthe oryzae* (MoMob1-XP_003716843.1).

*Aspergillus fumigatus* (AfMob1-XP_755571.1).

*Aspergillus nidulans* (AnMob1-XP_663892.1).

*Candida albicans* (CaMob1-XP_719093.1).

*Saccharomyces cerevisiae* (ScMob1-NP_012160.2).

*Schizosaccharomyces pombe* (SpMob1-NP_595191.1).

*Rhizoctonia solani* (RsMob1-XP_043179247.1).

## 3 Results

### 3.1 Identification of FgMob1 protein and generation of *FgMOB1* deletion mutant in *F. graminearum*

**Figure 1:**
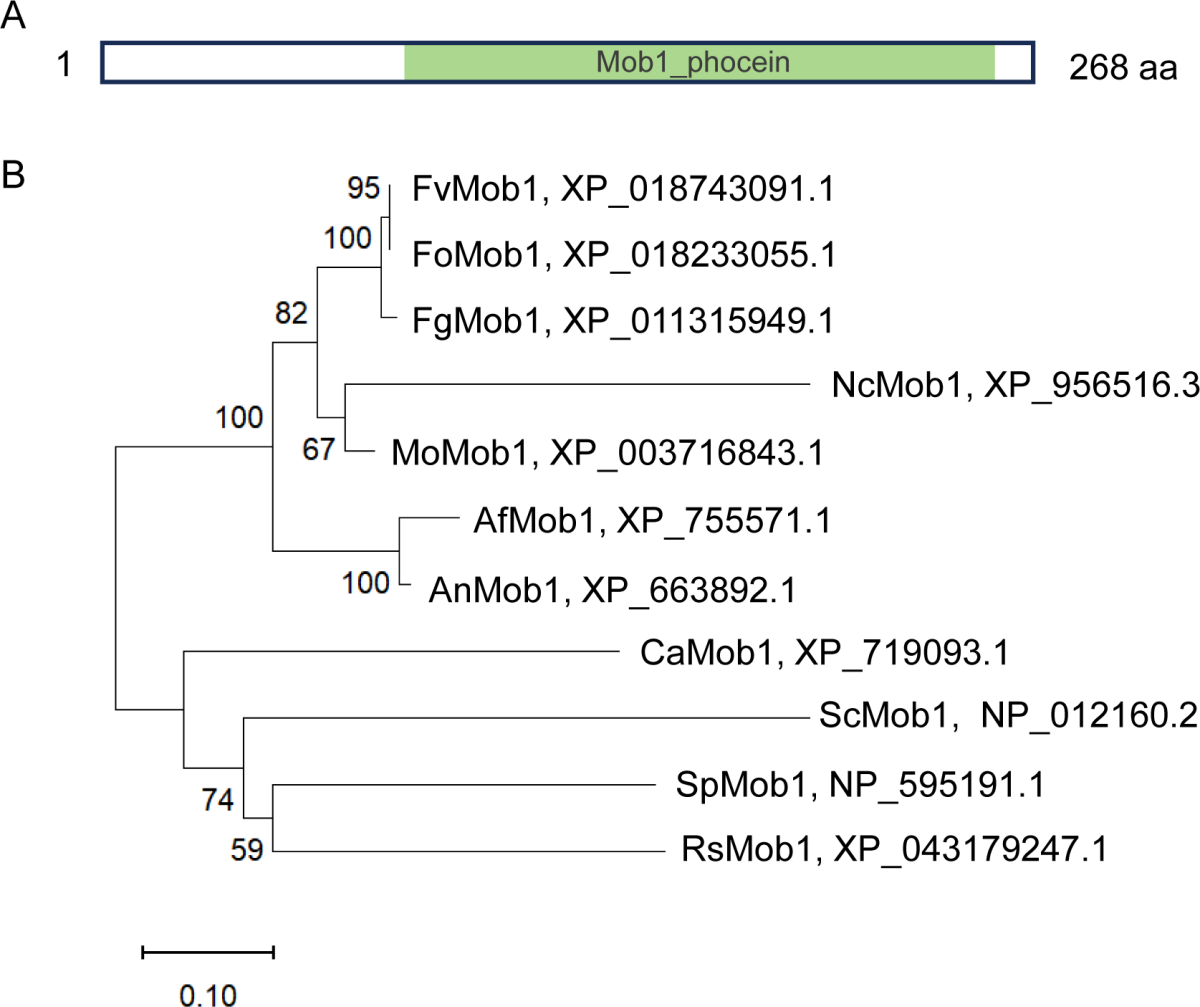
Phylogenetic analysis of Mob1 proteins from different species. **(A)** Conserved domain analysis of FgMob1. FgMob1 contains a single conserved Mob1_phocein domain. **(B)** Sequence alignments were performed using the Clustal X 1.83 program and the resulting phylogenetic tree was viewed using the MAGE 11 program. A neighbor-joining tree with 10,000 bootstrap replicates was generated for Mob1 homologs in different organisms.

We used a bioinformatics approach to identify the Mob1 protein, which belongs to the MOB (Mps One Binder) family in *F. graminearum*, as outlined in the materials and methods section 2.6. FgMob1 contains a conserved Mob1_phocein domain (**Figure 1A**). Phylogenetic analysis showed that FgMob1 is highly conserved in fungi, especially in *Fusarium oxysporum* and *Fusarium verticillioides* (**Figure 1B**).

To characterize the function of FgMob1, we generated a gene deletion mutant of *FgMOB1* using the split-marker approach (**Supplementary Figure 1A**) and selected ∆*Fgmob1-1*, ∆*Fgmob1*-*3*, and ∆*Fgmob1–4* transformants for Southern blot analysis, which showed a 1.7 kb band in the wild type PH-1 and a 3.1 kb band in the mutants (**Supplementary Figure 1B**). Furthermore, FgMob1 was tagged with GFP at its C-terminus under the control of its native promoter to form the FgMob1-GFP fusion vector. The constructed vector was then transformed into the protoplasts of the ∆*Fgmob1–4* mutant to generate the complemented strain ∆*Fgmob1*-*C*.

### 3.2 FgMob1 contributes to vegetative growth and aerial hyphal development in *F. graminearum*

**Figure 2:**
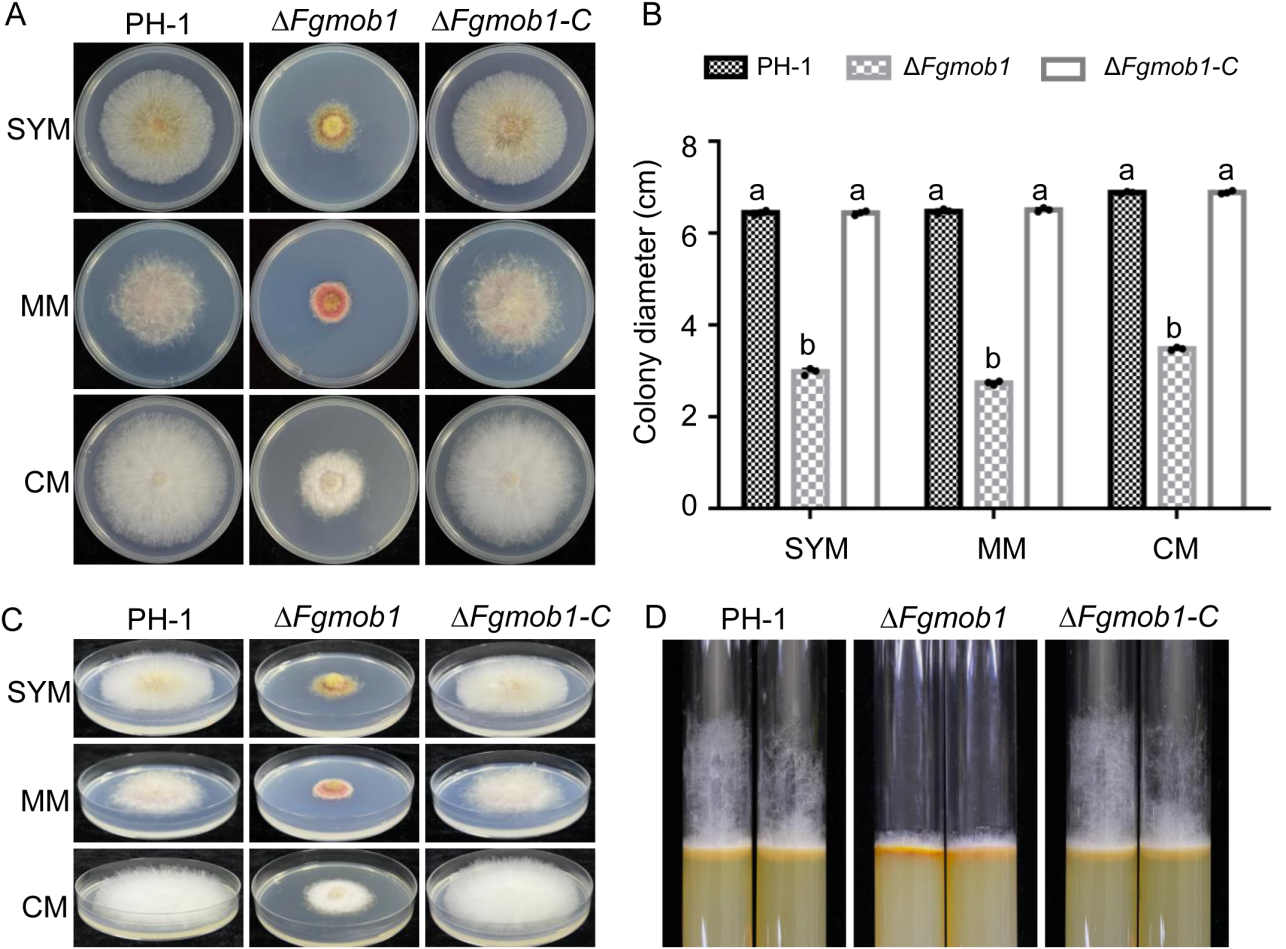
FgMob1 is required for colony morphology and vegetative growth in *Fusarium graminearum.* **(A, B)** Colony morphologies and diameters of the wild type (PH-1), the *FgMOB1* deletion mutant (Δ*Fgmob1*), and the complemented strain (Δ*Fgmob1*-C) grown on starch yeast medium (SYM), minimal medium (MM), and complete medium (CM) for 3 d (days). Photographs were taken 3 days after inoculation. Error bars represent mean ± SD from three replicates, and bars with the same letters are not significantly different at *P* < 0.05. **(C)** Aerial hyphae morphology of the Δ*Fgmob1* mutant on SYM, MM and CM agar plates. **(D)** Aerial hyphae morphology of the Δ*Fgmob1* mutant in a glass tube containing CM agar.

To determine whether FgMob1 is required for vegetative growth and colony morphology of *F. graminearum*, the wild type PH-1, the *FgMob1* deletion mutant (∆*Fgmob1*), and the complemented strain ∆*Fgmob1-C* were cultured on different media, including complete medium (CM), starch-yeast medium (SYM), and minimal medium (MM) for 3 days. As shown in **Figures 2A, B**, the ∆*Fgmob1* mutant exhibited significantly reduced vegetative growth on all three media compared to the PH-1 and ∆*Fgmob1-C* strains. Interestingly, the ∆*Fgmob1* mutant exhibited flattened aerial hyphae on these media (**Figure 2C**). Consistently, aerial hyphae of PH-1 and the ∆*Fgmob1* mutant were observed in glass tubes containing CM agar, and it was found that the length of the hyphae in the ∆*Fgmob1* mutant was significantly reduced compared to PH-1 (**Figure 2D**). Taken together, these results suggest that FgMob1 is important for vegetative growth and aerial hyphal development in *F. graminearum*.

### 3.3 FgMob1 plays a crucial role in asexual and sexual reproduction

**Figure 3:**
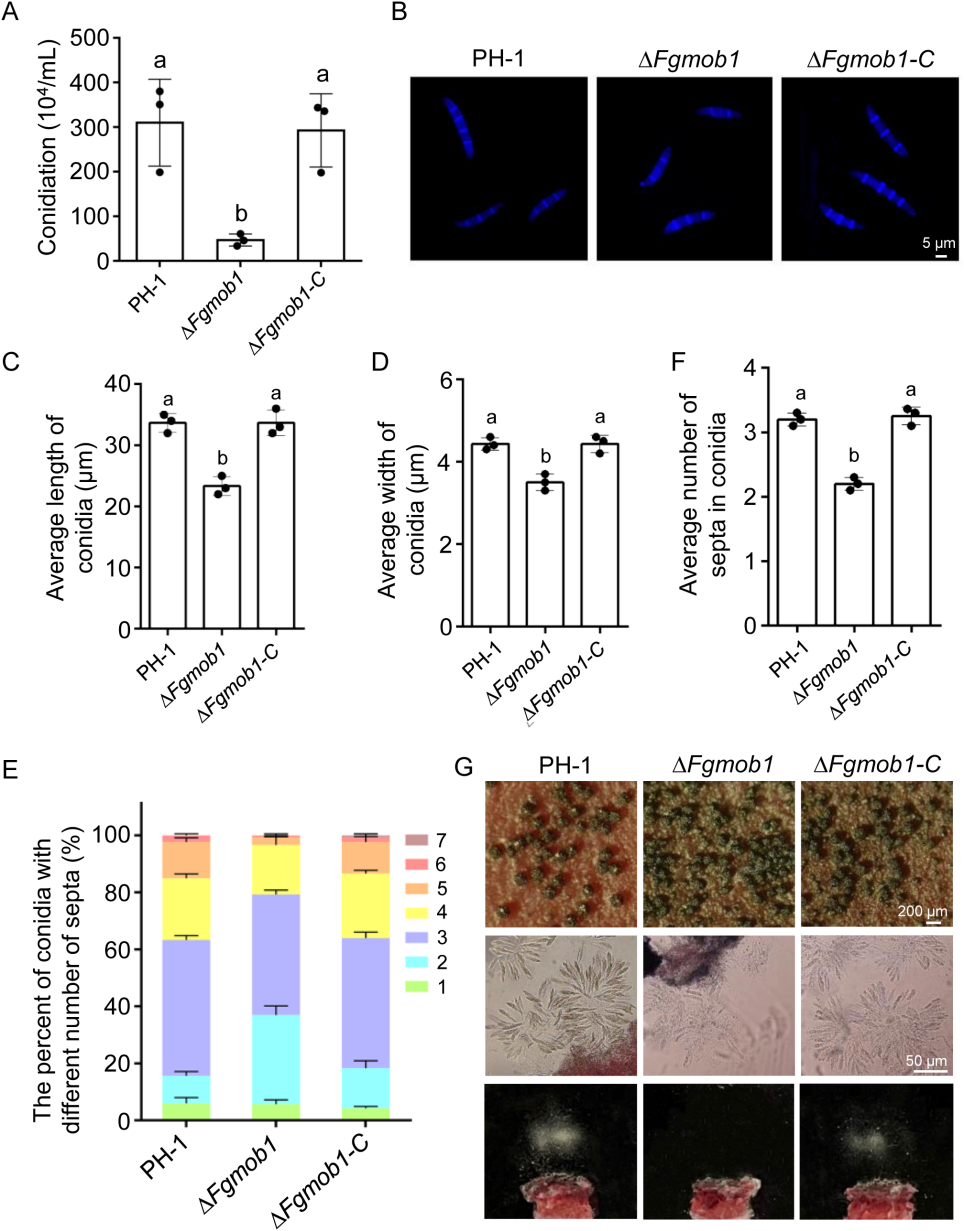
FgMob1 is involved in sexual and asexual development in *Fusarium graminearum*. **(A)** Conidiation of PH-1, Δ*Fgmob1* and Δ*Fgmob1*-*C* strains. Error bars represent mean ± SD from three replicates, and bars with the same letters are not significantly different at *P* < 0.05. **(B)** Conidial morphologies and number of septa in the PH-1, Δ*Fgmob1*, and Δ*Fgmob1-C* strains after incubation in liquid CMC for 3 days. Conidia were stained with 10 μg mL^-1^ calcofluor white (CFW). **(C, D)** Average lengths and widths of conidia from the PH-1, Δ*Fgmob1* and Δ*Fgmob1-C* strains. About 100 conidia were counted from each strain in each experiment. Error bars represent mean ± SD from three replicates, and bars with the same letters are not significantly different at *P* < 0.05. **(E)** Percentages of conidia with different numbers of septa in PH-1, Δ*Fgmob1*, and Δ*Fgmob1-C* strains. More than 100 conidia from each strain were counted in each experiment. Error bars represent standard deviations from three replicates. **(F)** Average number of septa in conidia of PH-1, Δ*Fgmob1* and Δ*Fgmob1-C* strains. **(G)** FgMob1 is not required for perithecia formation but is essential for ascospore maturation and release in *F. graminearum*.

Conidia are believed to play an important role in infecting flowering wheat heads in *F. graminearum*. To understand the function of FgMob1 in conidia formation, the PH-1, ∆*Fgmob1* and ∆*Fgmob1*-*C* strains were cultured in liquid CMC medium for 3 days to induce conidia production. Our results showed that conidiation in the *∆Fgmob1* mutant was drastically reduced compared to the PH-1 and *∆Fgmob1*-*C* strains (**Figure 3A**). Furthermore, we found that the conidia produced by the *∆Fgmob1* mutant were smaller than those produced by PH-1 and the complemented strain (**Figure 3B**); the average lengths and widths of conidia from the ∆*Fgmob1* mutant were significantly smaller than those in PH-1 and ∆*Fgmob1-C* strains (**Figures 3C, D**). In addition, the average number of septa per conidium in the ∆*Fgmob1* mutant was significantly lower than in the wild type PH-1 and ∆*Fgmob1-C* strains (**Figures 3E, F**). Calcofluor white (CFW) staining demonstrate that about 35% of the conidia produced by the ∆*Fgmob1* mutant contained fewer than two septa (**Figure 3E**), while only about 16% of the conidia produced by the wild type and complemented strains had this number of septa per conidium. Collectively, these results suggest that FgMob1 plays important roles in conidiation and conidial morphology of *F. graminearum*.

### 3.4 FgMob1 is important for pathogenicity and DON production

**Figure 4:**
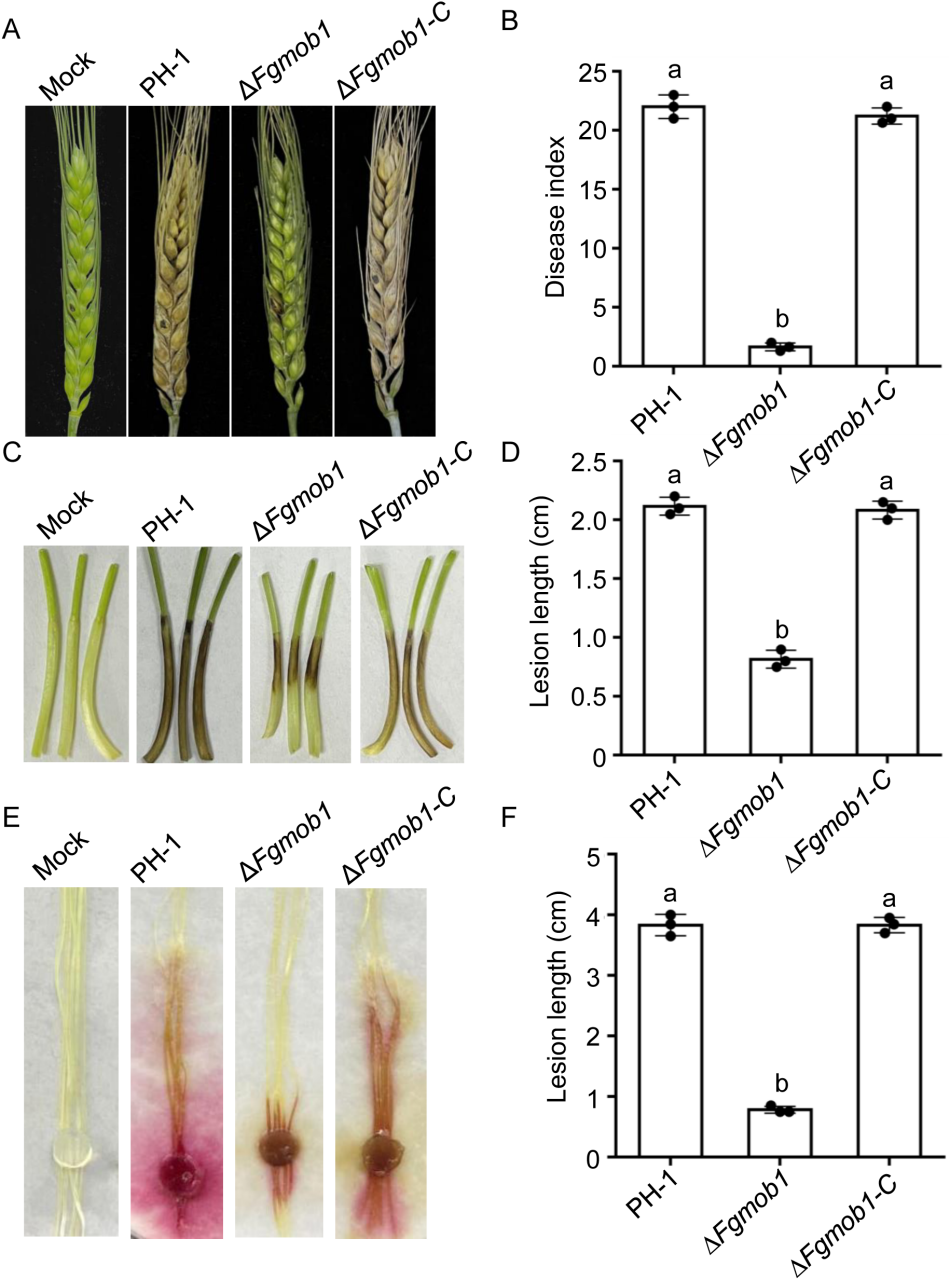
FgMob1 is important for the pathogenicity of *Fusarium graminearum*. **(A)** Pathogenicity of the wild type (PH-1), Δ*Fgmob1* and Δ*Fgmob1*-*C* strains inoculated on wheat heads for 14 days. The Δ*Fgmob1* mutant showed significantly reduced virulence. **(B)** Disease index rated based on the number of symptomatic spikelets. Error bars represent mean ± SD from three replicates, and bars with the same letters are not significantly different at *P* < 0.05. **(C, D)** Pathogenicity and lesion length of wild type PH-1 and Δ*Fgmob1* mutant inoculated on wheat coleoptiles for 7 d. Error bars represent mean ± SD from three replicates, and bars with the same letters are not significantly different at *P* < 0.05. **(E, F)** Pathogenicity and lesion length of PH-1 and Δ*Fgmob1* mutant inoculated on corn silks for 7 d. Error bars represent mean ± SD from three replicates, and bars with the same letters are not significantly different at *P* < 0.05.

Considering that *F. graminearum* is a devastating fungal pathogen of cereal crops, pathogenicity tests were conducted on wheat heads, wheat coleoptiles, and corn silks to determine the role of FgMob1 in the pathogenesis of *F graminearum*. As shown in **Figures 4A, B**, the wild type PH-1 and the complemented strain ∆*Fgmob1-C* were able to infect almost the entire wheat head and exhibited typical FHB symptoms, while the ∆*Fgmob1* mutant failed to spread to adjacent spikelets and caused only atypical head blight symptoms in the inoculated kernels (**Figures 4A, B**). Similar results were observed in the infection experiments on wheat coleoptiles and corn silks (**Figures 4C–F**); the lesion lengths caused by ∆*Fgmob1* on wheat coleoptiles and corn silks were significantly reduced compared to those in the wild type PH-1 and the complemented strain. Taken together, these results indicate that FgMob1 plays a crucial role in the pathogenicity of *F. graminearum*.

**Figure 5:**
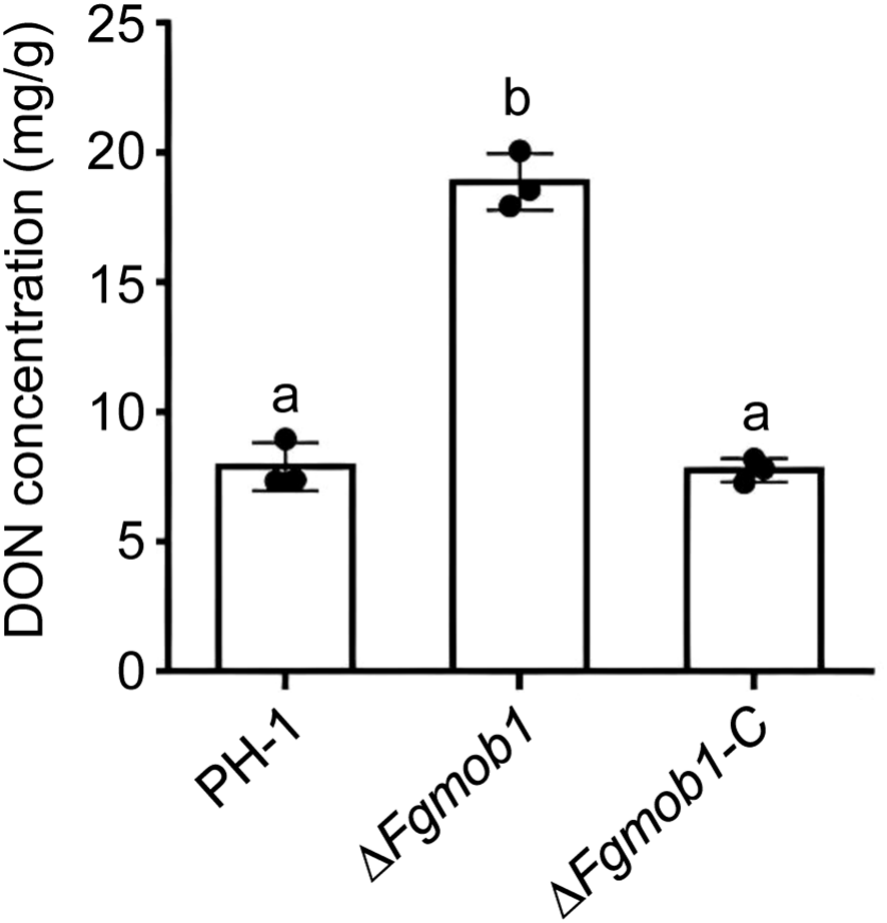
FgMob1 regulates the DON production in *Fusarium graminearum*. Deletion of the *FgMOB1* gene results in increased DON production in *F. graminearum*. Error bars represent mean ± SD from three replicates, and bars with the same letters are not significantly different at *P* < 0.05.

The mycotoxin deoxynivalenol (DON) is the most well-characterized virulence factor in *F. graminearum* (Proctor et al., 1995). To determine the role of FgMob1 in DON production, wild type PH-1, ∆*Fgmob1*, and ∆*Fgmob1-C* were inoculated in liquid trichothecene biosynthesis induction (TBI) medium for 7 days. As shown in **Figure 5**, DON production by the ∆*Fgmob1* mutant was significantly increased compared to that in PH-1 and ∆*Fgmob1-C* strains. Collectively, this result shows that FgMob1 negatively regulates DON production in *F. graminearum*.

### 3.5 Localization of FgMob1-GFP in *F. graminearum*

**Figure 6:**
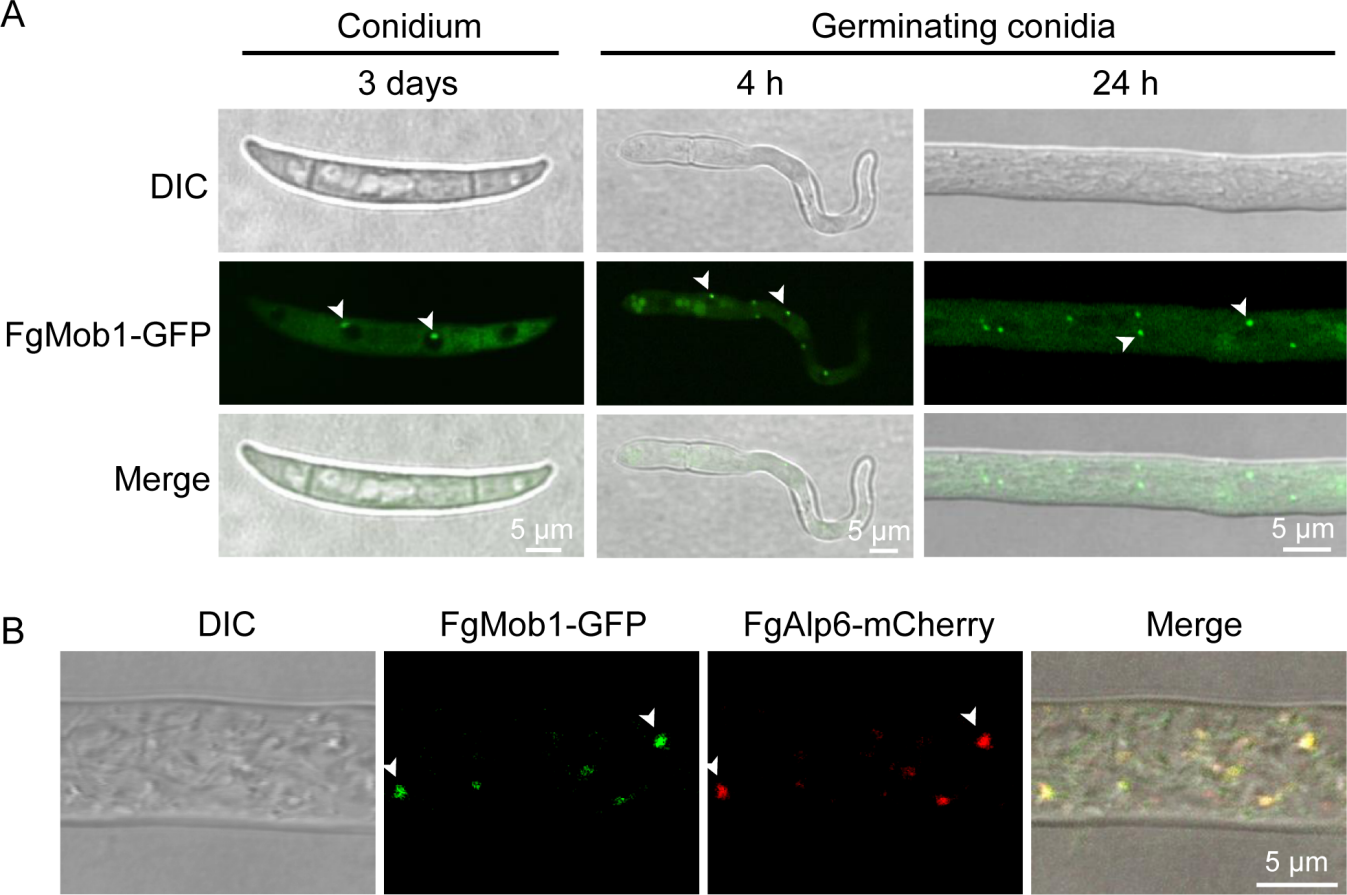
Localization of FgMob1-GFP in *Fusarium graminearum*. **(A)** FgMob1-GFP appears as punctate structures in the conidia (3 days), germinating conidia (4 h) and hyphae (24 h) of *F. graminearum*. White arrowheads indicate the localization of the punctate structures. **(B)** FgMob1-GFP colocalizes with the spindle pole body marker FgAlp6-mCherry. White arrowheads indicate the sites of co-localization.

To determine the subcellular localization of FgMob1 in *F. graminearum*, the complemented strain ∆*Fgmob1-C* was used, and the mycelia, conidia and germinating conidia of the strain were involved. Laser confocal microscopy revealed that FgMob1-GFP signals appear as punctate structures in mycelia, conidia and germinating conidia of *F. graminearum* (**Figure 6A**). Furthermore, to assess whether these punctate structures are spindle pole bodies (SPB), we co-transformed the FgMob1-GFP plasmid with the SPB marker FgAlp6-mCherry construct (Miao et al., 2023) into the protoplasts of wild type PH-1 and observed the GFP and mCherry signals. As shown in **Figure 6B**, FgMob1-GFP co-localizes with FgAlp6-mCherry in the fungal hyphae. This result indicates that FgMob1 localizes to the SPB in *F. graminearum.*

### 3.6 FgMob1 is involved in the mitotic exit network

**Figure 7:**
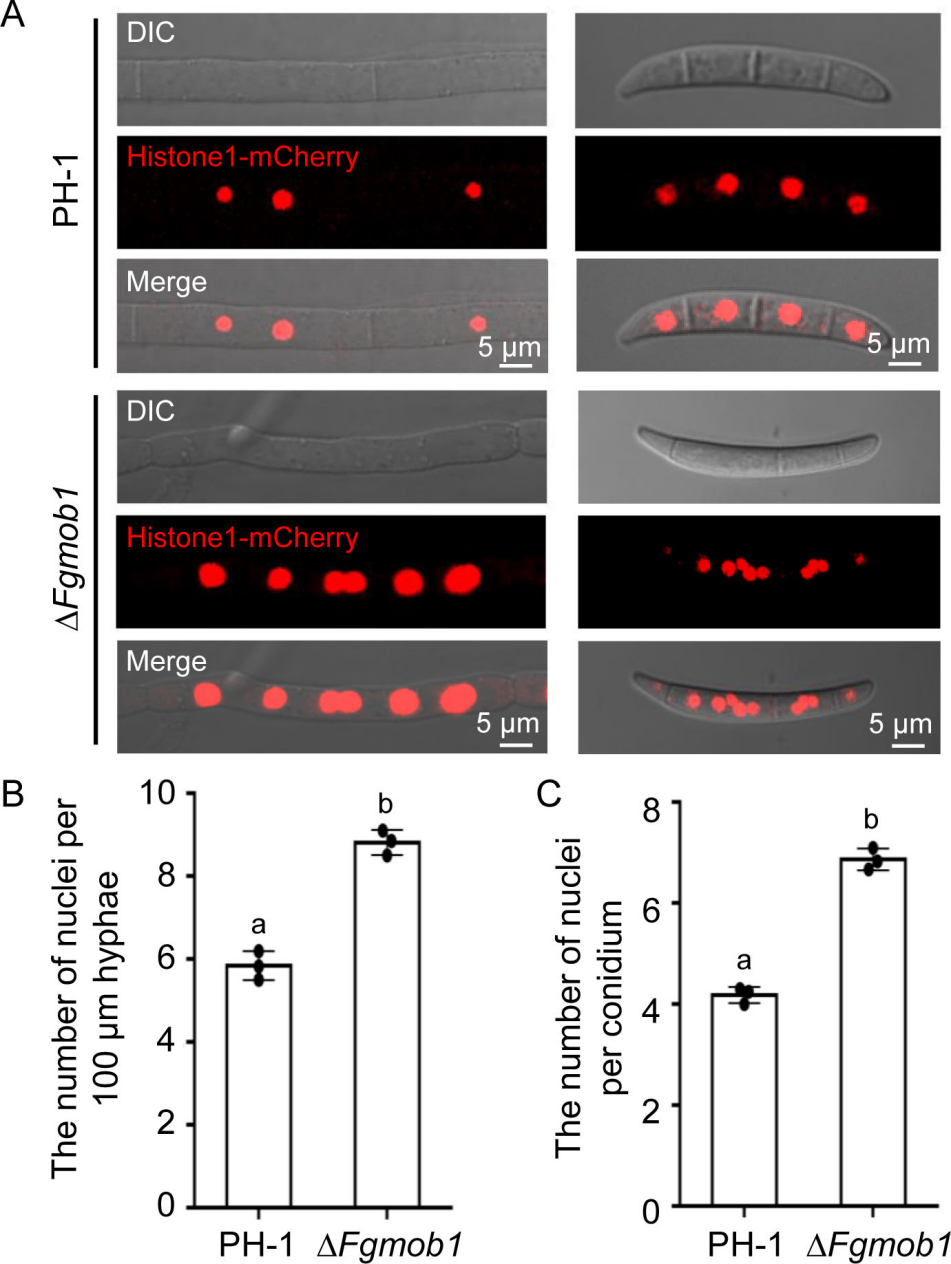
FgMob1 is involved in nuclear division. **(A)** The distribution of nuclei in the hyphae and conidia of PH-1 and Δ*Fgmob1* strains. Nuclei were distributed evenly along the hyphae in the wild type, while clumped nuclei were observed in the Δ*Fgmob1* mutant. **(B)** The number of nuclei per 100 μm of hyphae in the PH-1 and Δ*Fgmob1* strains. **(C)** The number of nuclei per conidium in PH-1 and Δ*Fgmob1* strains.

To test the conserved roles of FgMob1 in the MEN of *F. graminearum*, we introduced the Histone1-mCherry (H1-mCherry) fusion plasmid into the protoplasts of wild type PH-1 and the ∆*Fgmob1* mutant and observed them by confocal microscopy. As shown in **Figure 7A**, the number of nuclei was significantly increased in the hyphae and conidia of the ∆*Fgmob1* mutant compared to PH-1. On average, there are 5.84 nuclei per 100 μm of hyphae in PH-1, while an average of 8.87 nuclei per 100 μm of hyphae was observed in the ∆*Fgmob1* mutant (**Figure 7B**). Furthermore, we found that most of the conidia from PH-1 contain only one nucleus (rarely two) in each inter-septal compartment, whereas multiple nuclei were observed in each inter-septal compartment of the ∆*Fgmob1* mutant (**Figure 7A**). The average number of nuclei in the conidia of wild type PH-1 is 4.13, while it is 7.17 in the ∆*Fgmob1* mutant (**Figure 7C**). Taken together, these results indicate that FgMob1 negatively regulates nuclear division and is involved in MEN in *F. graminearum.*

### 3.7 FgMob1 is involved in the maintenance of cell wall integrity

**Figure 8:**
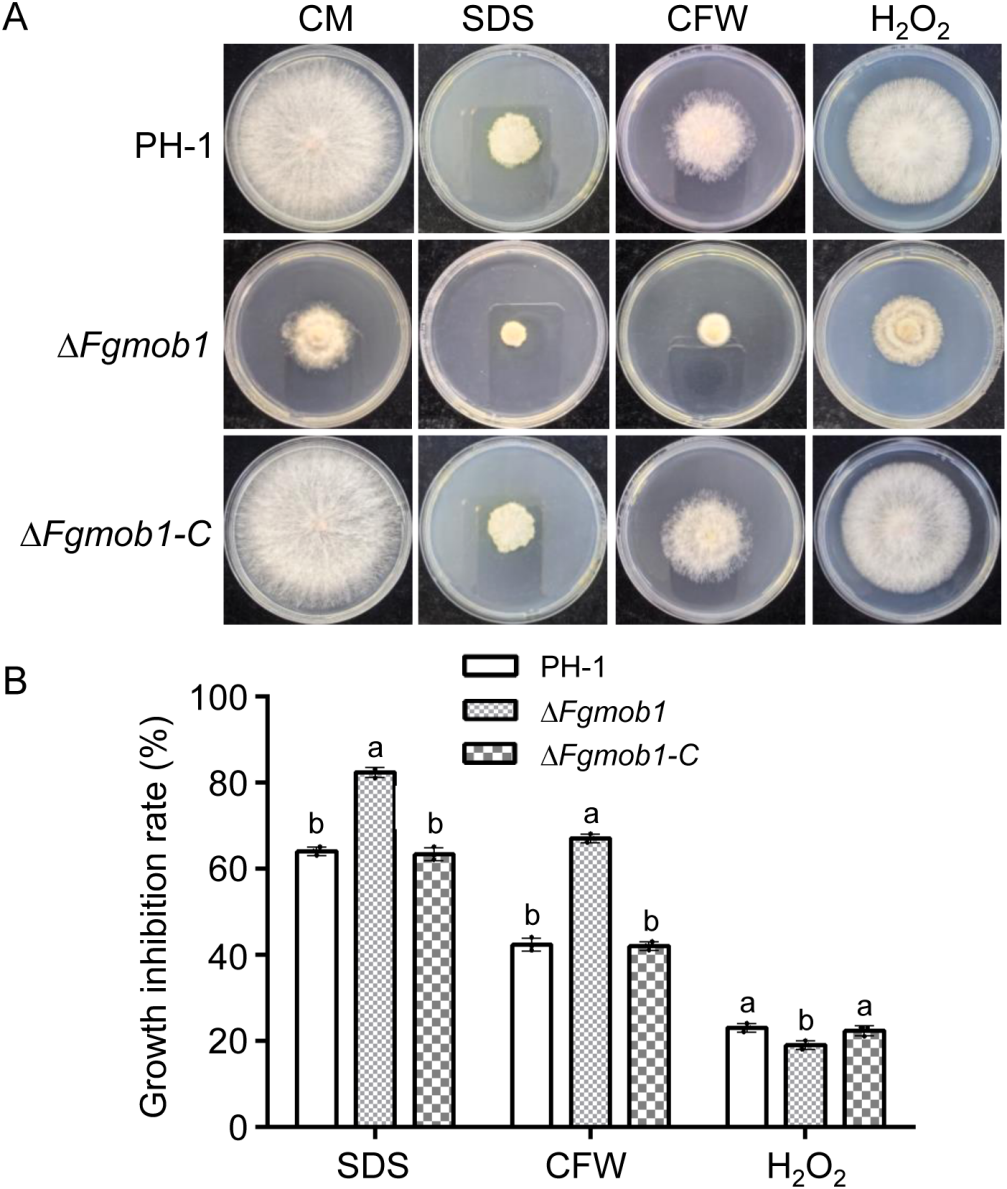
FgMob1 is involved in cell wall integrity. **(A)** The colony morphologies of PH-1, Δ*Fgmob1* and Δ*Fgmob1*-*C* strains grown on CM plates supplemented with SDS (0.02%), calcofluor white (CFW, 200 μg/mL), or H_2_O_2_ (0.03%). **(B)** Growth inhibition rates of PH-1, Δ*Fgmob1* and Δ*Fgmob1*-*C* strains grown on CM plates supplemented with SDS, CFW, and H_2_O_2_. The Δ*Fgmob1* mutant is more sensitive to SDS and CFW stresses. Error bars on each column denote the standard deviation of three repeated experiments. Different letters indicate a significant difference (*P* < 0.05).

The response to environmental or abiotic stresses is very important during *F. graminearum* infection of its host (Leplat et al., 2013). To investigate whether disruption of the MEN process affects the fungal response to environmental or abiotic stresses, mycelial plugs from fresh cultures of PH-1, ∆*Fgmob1*, and ∆*Fgmob1-C* strains were inoculated onto CM solid medium containing 0.02% SDS (sodium dodecyl sulfate, a cell membrane detergent), 200 µg/mL CFW (Calcofluor white, which is thought to interfere with cross-linking of the polysaccharide network), or 0.03% H_2_O_2_ (hydrogen peroxide, an oxidative stress-inducing agent). After 3 days, colony diameters were measured. As shown in **Figure 8A**, the wild type PH-1, ∆*Fgmob1*, and ∆*Fgmob1-C* strains all exhibited inhibited mycelia growth (**Figure 8A**). However, the mycelial inhibition rates of the ∆*Fgmob1* mutant to SDS and CFW were significantly increased compared to those in the PH-1 and ∆*Fgmob1-C* strains (**Figure 8B**). The mycelial inhibition rate of the ∆*Fgmob1* mutant to H_2_O_2_ was significantly decreased compared to the PH-1 and ∆*Fgmob1-C* strains. Collectively, these data suggest that FgMob1 regulates cell wall and membrane integrity as well as oxidative stress in *F. graminearum*.

## 4 Discussion

Mob proteins were first identified in yeast (Luca and Winey, 1998), and have since been found in a wide variety of eukaryotes (Luca and Winey, 1998; Duhart and Raftery, 2020). Mob1p is a core component of the mitotic exit network (MEN) and the septation initiation network (SIN) in budding and fission yeast, respectively (Gundogdu and Hergovich, 2019). The cell cycle is controlled by numerous mechanisms that ensure correct cell division (Miao et al., 2023). However, the roles of Mob1 homolog proteins in plant pathogenic fungi remain largely unclear. In this study, we showed that FgMob1 is required for vegetative growth, conidiation, DON production and pathogenicity in *F. graminearum*. Furthermore, FgMob1 localizes to the spindle pole bodies (SPB) and involved in the MEN. Additionally, deletion of the *FgMOB1* gene affects the response of *F. graminearum* to abiotic stresses.

In yeast, MEN is a signaling pathway driven by the GTPase Tem1, along with Cdc15, Dbf2, Dbf20, Mob1, Cdc14, Cdk1, and Sic1 (Hotz and Barral, 2014). In recent years, various studies have shown that MEN plays important roles in the development and pathogenicity of phytopathogens (Feng et al., 2021; Miao et al., 2023). The mitotic exit mediated by FgTem1 is essential for the pathogenicity of *F. graminearum* (Miao et al., 2023). FgCdc14 regulates cytokinesis, morphogenesis, and pathogenesis in *F. graminearum* (Li et al., 2015; Yun et al., 2015). Disruption of MEN results in defects in cell wall integrity (CWI) in *M. oryzae* (Feng et al., 2021); the ∆*Momob1* mutant was more susceptible to cell wall stress induced by Calcofluor white (CFW) and Congo red (CR). MoSep1-dependent MoMkk1 phosphorylation is required to balance cell division with CWI, maintaining the dynamic stability necessary for the pathogenicity of *M. oryzae* (Feng et al., 2021). CgMob1 is critical for maintaining CWI, and conferring resistance to the fungicide carbendazim in *Colletotrichum gloeosporioides* (Yang et al., 2025). The crystal structure of ScMob1 was identified, and it was found that the N-terminal region of Mob1 contains structural elements that are functionally important in yeast (Mrkobrada et al., 2006). In this study, we found that deletion of the *FgMOB1* gene also resulted in increased susceptibility to cell wall stress, suggesting a conserved role for the Mob1 protein in the CWI pathway in phytopathogens. Plants constantly encounter diverse biotic and abiotic stresses that threaten their survival (Ali et al., 2026). During *F. graminearum* infection of its host, sensitivity to environmental stresses is very important (Leplat et al., 2013). FgMob1 plays a significant role in the response to abiotic stress; deletion of the FgMob1 protein affects sensitivity to cell wall, plasma membrane, and oxidative stresses, suggesting that disruption of MEN in *F. graminearum* affects the response to abiotic stress. Reactive oxygen species (ROS) is a signaling molecule that modulate cellular homeostasis and defense responses during stress adaptation (Ali et al., 2025). FgMob1 may participate in redox signaling networks and thereby establish a critical link between cell cycle control and stress adaptation. FgMob1, as a conserved component of the MEN, primarily regulates the Dbf2 kinase in yeast, which in turn activates the Cdc14 phosphatase to promote mitotic exit (Stegmeier et al., 2002; Visintin et al., 2003; Kao et al., 2014). Dbf2 and Cdc14 both involved in septation or cytokinesis in *M. oryzae* (Li et al., 2018; Feng et al., 2021), *F. graminearum* (Li et al., 2015; Du et al., 2026), and *C. gloeosporioides* (Yang et al., 2025). Cytokinesis is the final step of the cell cycle, resulting in the generation of two progeny; failure of correct cell division may be lethal for both mother and daughter cells (Walther and Wendland, 2003). FgMob1 localizes to spindle pole bodies and negatively regulates nuclear division. Loss of this regulation may uncouple mitotic exit from actomyosin ring contraction and septum formation, leading to excess nuclei arising from failed cell division following multiple nuclear cycles. Previous studies demonstrated that the Dbf2-Mob1 complex functions downstream of spindle position checkpoints, dysregulation of this complex uncouples nuclear division from cytokinesis, resulting in the formation of multinucleate cells (Stoepel et al., 2005). Without proper MEN signaling, Cdc14 is not fully released from the nucleolus, preventing cyclin-dependent kinase (Cdk) inactivation and causing cells to arrest in late anaphase with divided but decondensed nuclei (Mohl et al., 2009).

The perithecium produced by sexual reproduction is important for overwintering in the life cycle of *F. graminearum* (Bushnell et al., 2003; Trail, 2009). The non-pheromone receptor Gip1 regulates two distinct sexual differentiation processes during perithecial development (Ding et al., 2025). The *gia1* mutant is normal in perithecium development, crozier formation, and karyogamy but fails to undergo meiosis (Ding et al., 2023). In this study, we found that perithecia could form normally, while ascospore formation was dramatically affected and asci were abnormal and immature in the *FgMOB1* deletion mutant on carrot agar medium. The ∆Fgmob1 mutant shows a similar phenotype in sexual development to the *gia1* mutant, suggesting that disruption of the *FgMOB1* gene may affect the capacity to undergo meiosis in *F. graminearum*.

The *F. graminearum* genome contains three Mob proteins (Liu et al., 2021). FgMob2 and FgMob3 were reported in previous studies (Liu et al., 2021; Chen et al., 2023). FgMob2 interacts with FgCot1 and is critical for polarity, fungal development, cell wall organization, lipid metabolism and virulence in *F. graminearum* (Liu et al., 2021). The STRIPAK complex component FgMob3 orchestrates cell wall integrity signaling to govern fungal development and virulence (Chen et al., 2023). Here, we show that FgMob1 is involved in MEN and regulates vegetative growth, aerial hyphal development, cell wall integrity, conidiation, DON production and pathogenicity. Together with previous reports (Liu et al., 2021; Chen et al., 2023), we find that all three Mob proteins (FgMob1, FgMob2, and FgMob3) are critical for fungal development and virulence in *F. graminearum*.

DON biosynthesis is significantly increased in the ∆*Fgmob1* mutant, indicating that FgMob1 negatively regulates DON production. DON production is one of the major virulence factors in *F. graminearum* (Proctor et al., 1995). Oxidative burst is a common plant defense response, and ROS have been shown to induce DON production (Jiang et al., 2015). FgMkk1 functions upstream of Mgv1 and regulates multiple stress responses and virulence via both the CWI and HOG pathways (Yun et al., 2014). Nuclear accumulation and division defects resulting from FgMob1 deficiency may promote DON upregulation through CWI and oxidative stress responses. As a core regulator of MEN, FgMob1 also may directly couple cell−cycle checkpoint signals to transcriptional activation of the *TRI* genes. The reduced virulence of the ∆*Fgmob1* mutant toward its host may be due to its slow growth rate and high sensitivity to abiotic stress. In addition, we have found some gene deletion mutants that grow slowly and are important for pathogenicity, while DON production is significantly increased in previous studies (Zheng et al., 2021; Wu et al., 2022), suggesting that DON is not the sole virulence factor.

## Data Availability

The original contributions presented in the study are included in the article/**Supplementary Material**. Further inquiries can be directed to the corresponding authors.

## Supplementary material

Supplementary Figure 1 Southern blot analysis of *FgMOB1* gene deletion mutants. **(A)** Split-marker approach for the deletion of *FgMOB1* gene. The gene was deleted from PH-1 background. **(B)** Southern blot of wild type PH-1 and *FgMOB1* deletion mutants, *Xho*I (X) digested DNAs showed a 1.7 kb in the WT and a 3.1 kb in mutants, the black and red asterisks represent the correct bands for the WT and ∆*Fgmob1* mutant, respectively.
