# Supplementary material for: FgMob1 is involved in mitotic exit and regulates the development, abiotic stress response, and pathogenicity of Fusarium graminearum
Source: Front Plant Sci. 2026 Jun 11;17:1871251. doi: 10.3389/fpls.2026.1871251 (PMC13294386; doi:10.3389/fpls.2026.1871251)
Supplement: Supplementary Figure 1 — Southern blot analysis of FgMOB1 gene deletion mutants. (A) Split-marker approach for the deletion of FgMOB1 gene. The gene was deleted from PH-1 background. (B) Southern blot of wild type PH-1 and FgMOB1 deletion mutants, XhoI (X) digested DNAs showed a 1.7 kb in the WT and a 3.1 kb in mutants, the black and red asterisks represent the correct bands for the WT and ∆Fgmob1 mutant, respectively. [file DataSheet1.docx]

Supplementary Material

# Supplementary Figures and Tables

## Supplementary Figures


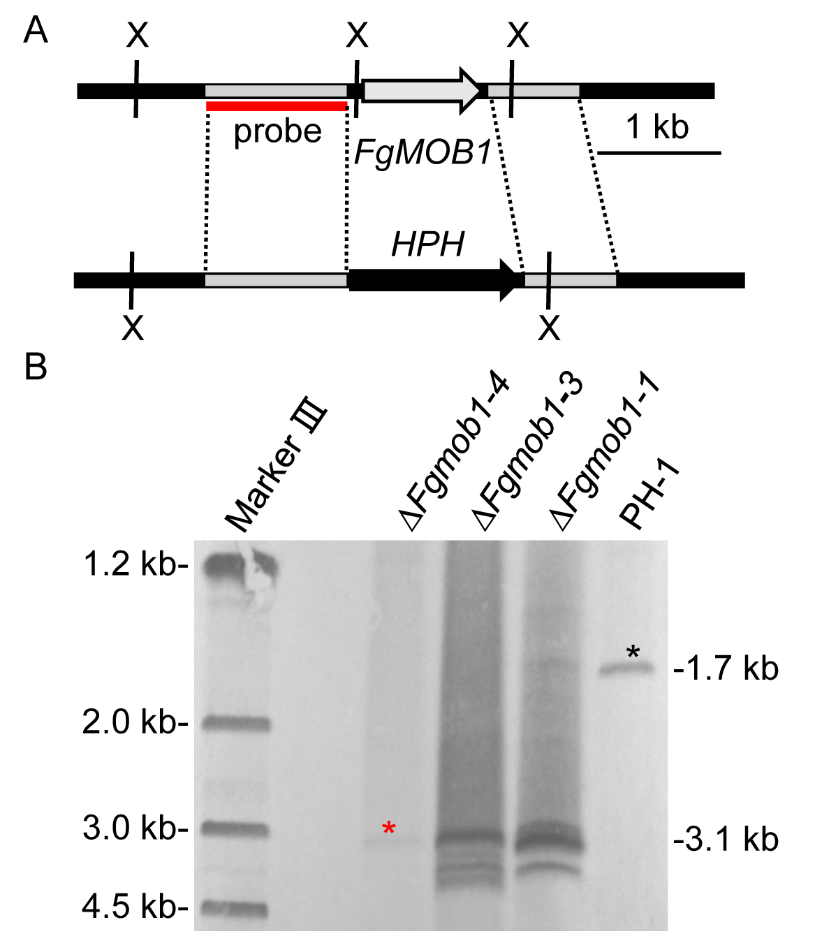


**Supplementary Figure 1** **Southern blot analysis of *FgMOB1* gene deletion mutants.**

(A) Split-marker approach for the deletion of the *FgMOB1* gene. The gene was deleted from the PH-1 background. (B) Southern blot of wild type PH-1 and *FgMOB1* deletion mutants. *Xho*I (X)-digested DNAs showed a 1.7 kb band in the wild type and a 3.1 kb band in the mutants, the black and red asterisks represent the correct bands for the WT and ∆*Fgmob1* mutant, respectively.

## Supplementary Tables

**Supplementary Table S1** Wild type (PH-1) and mutant strains used in this study.

| **Strain** | **Genotype description** | **Reference** |
| --- | --- | --- |
| PH-1 | Wild type | ([Cuomo *et al.*, 2007](#_ENREF_1)) |
| Δ*Fgmob1* | FGSG_00301 gene deletion in PH-1 | This study |
| Δ*Fgmob1-C* | Δ*Fgmob1* strain expressing the pFgMob1-GFP construct | This study |
| PH-1+Histone1-mCherry | PH-1 strain expressing the Histone1-mCherry construct | This study |
| Δ*Fgmob1*+Histone1-mCherry | Δ*Fgmob1* strain expressing the Histone1-mCherry construct | This study |
| PH-1+FgMob1-GFP+FgApl6-mCherry | PH-1 strain expressing the FgMob1-GFP and FgAlp6-mCherry constructs | This study |

**Supplementary Table S2** PCR primers used in this study.

| **Primer** | **Sequence (5΄→3΄)** | **Application** |
| --- | --- | --- |
| *FgMOB1-*AF | TATGTGACCTCCAACCCTT | *FgMOB1* deletion |
| *FgMOB1-*AR | TTGACCTCCACTAGCTCCAGCCAAGCCCGATGCTGCTACATTCCA |  |
| *FgMOB1-*BF | GAATAGAGTAGATGCCGACCGCGGGTTAGCAACGCAACAAGGAGT |  |
| *FgMOB1-*BR | TGACCAGCTAATTGACAACAC |  |
| *FgMOB1-*OF | GTAACCAGCGAACGAGAAA |  |
| *FgMOB1-*OR | AGTAAATGTGGGCGTAGACA |  |
| *FgMOB1-*UA | CGCCCTCAGCCCAAGAAGA |  |
| H853 | GACAGACGTCGCGGTGAGTT |  |
| YG/F | GATGTAGGAGGGCGTGGATATGTCCT |  |
| HY/R | GTATTGACCGATTCCTTGCGGTCCGAA |  |
| HYG/F | GGCTTGGCTGGAGCTAGTGGAGGTCAA |  |
| HYG/R | AACCCGCGGTCGGCATCTACTCTATTC |  |
| *FgMOB1-*CF | agggaacaaaagctgggtaccGCAGCCGAGTATGTTATGG | complementation |
| *FgMOB1-*CR | GCCGCCGCCGCCGCCAAGCTTGTCGCTCTTGAGCATGCTAT |  |

**REFERENCES**

Cuomo, C.A., Gueldener, U., Xu, J.R., Trail, F., Turgeon, B.G., Di Pietro, A., et al. (2007). The Fusarium graminearum genome reveals a link between localized polymorphism and pathogen specialization. Science 317, 1400-1402. doi: DOI 10.1126/science.1143708
